# Influenza Anti-Stalk Antibodies: Development of a New Method for the Evaluation of the Immune Responses to Universal Vaccine

**DOI:** 10.3390/vaccines8010043

**Published:** 2020-01-24

**Authors:** Alessandro Manenti, Agnieszka Katarzyna Maciola, Claudia Maria Trombetta, Otfried Kistner, Elisa Casa, Inesa Hyseni, Ilaria Razzano, Alessandro Torelli, Emanuele Montomoli

**Affiliations:** 1 VisMederi Research s.r.l., 53100 Siena, Italy; alessandro.manenti@vismederiresearch.com (A.M.); agnieszka.maciola@vismederiresearch.com (A.K.M.); casa@vismederiresearch.com (E.C.); hyseni@vismederiresearch.com (I.H.); ilaria.razzano@vismederiresearch.com (I.R.); montomoli@vismederi.com (E.M.); 2 VisMederi s.r.l., 53100 Siena, Italy; kistner@vismederi.com; 3 Department of Molecular and Developmental Medicine, University of Siena, 53100 Siena, Italy

**Keywords:** hemagglutinin, stalk domain, HA2-antibody, competitive ELISA, universal influenza vaccine

## Abstract

Growing interest in universal influenza vaccines and novel administration routes has led to the development of alternative serological assays that are able to detect antibodies against conserved epitopes. We present a competitive ELISA method that is able to accurately determine the ratio of serum immunoglobulin G directed against the different domains of the hemagglutinin, the head and the stalk. Human serum samples were treated with two variants of the hemagglutinin protein from the A/California/7/2009 influenza virus. The signals detected were assigned to different groups of antibodies and presented as a ratio between head and stalk domains. A subset of selected sera was also tested by hemagglutination inhibition, single radial hemolysis, microneutralization, and enzyme-linked lectin assays. Pre-vaccination samples from adults showed a quite high presence of anti-stalk antibodies, and the results were substantially in line with those of the classical serological assays. By contrast, pre-vaccination samples from children did not present anti-stalk antibodies, and the majority of the anti-hemagglutinin antibodies that were detected after vaccination were directed against the head domain. The presented approach, when supported by further assays, can be used to assess the presence of specific anti-stalk antibodies and the potential boost of broadly protective antibodies, especially in the case of novel universal influenza vaccine approaches.

## 1. Introduction

Influenza continues to have a significant impact on public health and is still responsible for high morbidity and mortality in humans, with annual attack rates estimated to be up to 10% in adults and 30% in children [1]. Vaccination is still the most effective method of preventing the morbidity and mortality caused by influenza infection, especially in groups at high risk of dangerous complications, such as young children and the elderly [2], although the effectiveness of influenza vaccination is strictly dependent on the age-group and vaccine formulation [3,4]. Influenza A and B viruses, which are responsible for annual epidemics in humans, undergo antigenic changes within the antibody-binding sites of the hemagglutinin (HA) and neuraminidase (NA) antigens; these changes are able to render the new strains different enough to at least partially avoid the immunity induced by previous infection or vaccination (antigenic drift) [5,6]. Consequently, the composition of vaccines needs to be updated every year in response to changes in HA antigens. Current inactivated intramuscular/intradermal vaccines (IIVs) or live attenuated influenza vaccines (LAIVs) are made with a carefully standardized amount of HA from three (trivalent influenza vaccine—TIV) or four (quadrivalent influenza vaccine—QIV) seasonal strains on the basis of recommendations by the World Health Organization (WHO) [7,8]. Despite the efforts of the WHO Collaborating Centers and the new mathematical modelling approach [6,9] to monitor antigenic drift, an intrinsic uncertainty concerning the match between the circulating viruses and the vaccine strains remains [10]. 

Another important consideration is the fact that the currently available influenza vaccines are not able to protect against emerging pandemic-like influenza viruses [11]. Moreover, with today’s manufacturing technologies, it would take at least six-to-eight months to prepare a new vaccine; in the event of urgent necessity, this may be too long, as demonstrated by the 2009 H1N1 pandemic [12,13].

The development of a universal influenza vaccine would avoid potential mismatches of recommended vaccine strains and the need for the annual re-formulation and re-administration of vaccines; it would also enable timely intervention in the event of a pandemic, and it might result in the eradication of influenza B virus in humans. Several candidate target antigens could be considered for use in universal influenza vaccines, such as the M2 ion channel [14], NA [15], and conserved regions of the head domain (HA1) [16] and stalk domain (HA2) of HA [17]. The stalk domain is the most conserved region of HA in the influenza A and B viruses. Its main function is to mediate the fusion of the viral and endosomal membranes once the virus has been internalized by endosomes in order to permit the release of the viral genome into the cytosol [18]. In order to carry out this function, the stalk domain has to undergo considerable structural rearrangements; this is why all possible mutations that could potentially interfere with this process are not permitted [17]. 

Classically, antibody-mediated immune responses after influenza vaccination or natural infection are assessed by standard serological assays such as hemagglutination inhibition (HI), single radial hemolysis (SRH), and micro-neutralization (MN) [19]. These methods are recommended by regulatory authorities and are considered the gold standard in detecting the immune response in serum samples. The HI assay detects antibodies that bind to the viral HA and prevent the agglutination of red blood cells (RBCs) by blocking the receptor binding site. The MN assay identifies functional neutralizing antibodies, including those that recognize epitopes in the stalk region of HA, which are conserved among different subtypes of influenza A viruses. The SRH assay may recognize not only antibodies against the surface glycoproteins but also those against the internal antigens [20]. However, these assays are generally insufficient to detect the immune response after immunization with LAIVs or conserved epitope-based vaccines. Moreover, HI titers are not always able to predict the right degree of protection from a disease, especially in children [21], the elderly [22] and obese subjects [23].

The growing interest in developing a universal influenza vaccine has led to the need for alternative serological assays that are able to detect different classes of antibodies, such as anti-stalk, anti-NA, and secretory immunoglobulin A (s-IgA) ones [24]. Stalk-specific antibodies can be detected mainly by enzyme-linked immunosorbent assays (ELISAs) by using purified chimeric (cHA) proteins, such as cH6/1 (which contains an H6 head domain from A/mallard/Sweden/81/02 combined with an H1 stalk domain of A/California/04/09) [25].

ELISAs, including the competitive assay described in the present study, are not able to predict whether the antibodies detected are functional. In order to support the results of ELISA, other assays can be adapted on the basis of some functions that anti-stalk antibodies can exert through various mechanisms, such us neutralization, Fc receptor activation, and NA inhibitory activity [26].

In this paper, we present a potential method of indirectly detecting specific anti-stalk serum immunoglobulin G (IgG) antibodies against conserved epitopes among group 1 and group 2 influenza A viruses by measuring the difference between the HA head and total HA response; this method, based on a re-adaptation of a competitive ELISA, allows for the discrimination and the quantification of antibodies that are directed against the head and stalk subunits. The construction of a stable headless HA would be an important step both for universal vaccine studies and serological assay use. There have been several studies [27,28,29] that were animated by the quest to find a stable form of an HA stalk without the head domain, but the correct stabilization and folding of the constructs remain to be evaluated in more detail. In this study, we evaluate the performance of the assay by measuring anti-head and anti-stalk responses in a small panel of human serum samples (adults and children) taken before and after vaccination in the 2009/2010 season.

## 2. Materials and Methods

### 2.1. Virus Antigen

The virus antigen and infectious influenza virus was the seasonal influenza strain A/California/7/2009 H1N1 (15/252), grown in eggs and obtained from NIBSC, UK.

### 2.2. Pseudotype Production

Lentiviral pseudo-virus particles (PVs) were produced by co-transfecting Human Embryonic Kidney (HEK) 293T/17 (ATCC® CRL-11268™) cells with phCMV1-H11 (H11 from A/ruddy turnstone/New Jersey/650653/2002 (H11N9)) and pI.18-N1Cal/09, as previously described. The H11 plasmid was added to make the NA more stable and to increase PV release and production. Briefly, 1 µg of HA, 1 µg of NA and 1.5 µg pNLLuc4.3 plasmids were transfected into HEK293T/17 cell lines by means of Endofectin™ Lenti (3µL/µg). The medium was changed 24 hours after transfection, and PVs were harvested after 48 hours. The titration of the NA activity of each PV was performed in an enzyme-linked lectin assay (ELLA), as in the protocol reported by Biuso et al. [30].

### 2.3. Serum Samples 

Human serum samples (*n* = 48; obtained before and after vaccination) were kindly provided by the Laboratory of Molecular Epidemiology, Department of Molecular and Developmental Medicine, University of Siena, where they had been stored in compliance with Italian ethics law. The following information was available for each serum sample: adult (18+ years) or child (3–9 years) age-group, year of sampling (2009–2010), and pre- and post-vaccination withdrawal.

### 2.4. Hemagglutination Inhibition Assay 

Serum samples were pre-treated with a receptor-destroying enzyme (RDE—Denka Seiken) for 18 hours at 37 °C in a water bath and then heat-inactivated for 1 hour at 56 °C in a water bath. At the end of incubation, all serum samples were treated with a 10% turkey RBCs (TRBCs) solution in order to remove non-specific inhibitors, and they were run in the HI assay by using the A/California/7/2009 H1N1pdm09 influenza strain, as described elsewhere [31]. HI titers below 10 were assigned a titer of 5 and considered negative.

### 2.5. Single Radial Hemolysis Assay

Serum samples were heat-inactivated at 56 °C for 30 minutes in a water bath before testing. Then, 6 µL of each serum sample was tested in SRH plates that were prepared in accordance with the protocol described by Trombetta and colleagues [32] in which the virus antigen was diluted at 2000 hemagglutinin units per milliliter in a TRBC suspension and guinea pig complement. The diameters of hemolysis were read in millimeters by a dedicated calibrating viewer.

### 2.6. Micro-Neutralization Assay

The MN assay was performed as described previously [33]. Briefly, heat-inactivated serum samples were mixed and incubated for 1 hour at 37 °C and 5% CO_2_ in a humidified atmosphere with a standardized amount of live A/California/7/2009 H1N1 influenza virus (100 tissue culture infective dose 50% (TCID50)). After the incubation period, the serum–virus mixtures were transferred to a plate that contained 90% confluent pre-seeded Madin–Darby canine kidney (MDCK) (ATCC® CCL-34™) cells that were monolayered in an UltraMDCK serum-free medium (Lonza, Milano, Italy) with 7 µg/ml of acetylated trypsin (Sigma, St. Louis, MO, USA). The plates were then incubated for 5 days at 37 °C and 5% CO_2_ in humidified atmosphere before being inspected by an inverted optical microscope for the presence/absence of a cytopathic effect (CPE).

### 2.7. Enzyme-Linked Lectin Assay 

Anti-NA antibodies were also determined by the ELLA assay in accordance with the protocol described by Couzens and colleagues [34]. Briefly, inactivated and 2-fold diluted serum samples were mixed with a standardized amount of influenza pseudotypes bearing N1 from A/California/7/2009, and incubated for 16–18 hours in a fetuin- (Sigma, St. Louis, MO, USA) coated plate. After the incubation period, the plates were washed, and peanut agglutinin (PNA) that was conjugated to horse-radish peroxidase (HRP) (Sigma, St. Louis, MO, USA) was added to all wells. After 2 hours of incubation, the plates were washed, and an o-phenylenediamine dihydrochloride (OPD) (Sigma, St. Louis, MO, USA) substrate was added. The reaction was stopped, and the absorbance was read at 490 nm.

### 2.8. Competitive ELISA for Anti-HA2 Antibody Detection

**Figure 1:**
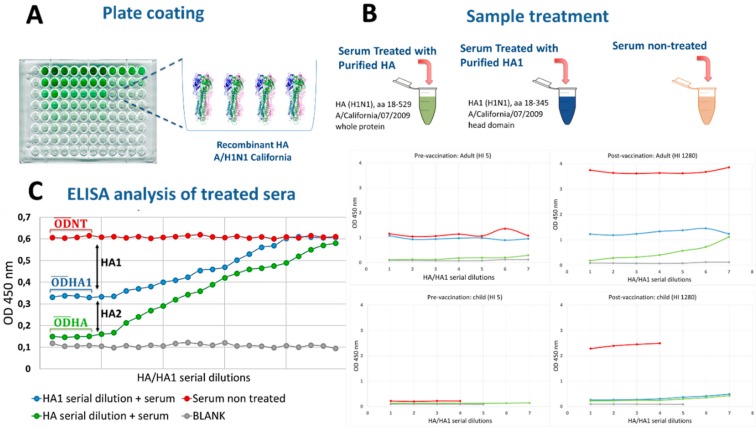
Schematic overview of the competitive ELISA method. (**A**) ELISA plates were coated with a purified hemagglutinin (HA) recombinant protein from A/California/7/2009 (H1N1) influenza strain. (**B**) A 1:250 pre-diluted serum sample is treated and incubated with different HA and HA1 concentrations. (**C**) The resulting OD difference between the highest head domain (HA1)-treated and the HA-treated sample can be attributed to the stalk domain (HA2) response. Two examples of treatment are reported, with appreciable pre-vaccination differences in the HA2 response between adults and children. $\bar{\mathrm{OD}}\mathrm{HA}1\;$ is the average optical density (OD) of the samples that were incubated with the head protein (HA1); $\bar{\mathrm{OD}}\mathrm{HA}\;$is the average OD of the samples that were incubated with the HA protein; and $\bar{\mathrm{OD}}\mathrm{NT}\;$is the average OD of the samples that were incubated with the buffer (non-treated samples).

The competitive ELISA procedure described here (Figure 1) utilized the ELISA Starter Accessory Kit (Bethyl Laboratories, Montgomery, TX, USA). ELISA plates were coated with purified recombinant HA (aa 18–529) (eEnzyme, Gaithersburg, MD, USA); serum samples were incubated with purified recombinant HA (aa 18–529) and head (aa 18–345) proteins from the A/California/7/2009 H1N1 influenza virus (eEnzyme, Gaithersburg, MD, USA). A solution of 5% non-fat dried milk (NFDM; Euroclonelone, Pero, Italy) in 0.05% Tris buffered saline-Tween 20 (TBS-T) (Thermo Scientific, Rodano, Italy) was used for plate blocking. ELISA 96-well plates were coated with the HA protein at a concentration of 1 µg/mL and incubated overnight at 4 °C. For each serum sample tested, three incubation conditions were prepared: 1) the HA recombinant protein in serial dilutions; 2) the head recombinant protein in serial dilutions; and 3) the TBS-T buffer without a protein, which was used for treatment control. Series of two-fold dilutions of HA and head proteins in TBS-T were prepared in rows of dedicated 96-well dilution plates. The starting concentration of the protein was 75 µg/mL in the first well, each well containing a volume of 20 µL of the solution. For each sample tested, one control row of wells containing 20 µL of the buffer (without HA or head proteins) was prepared. Serum samples that were designated for treatment were pre-diluted in TBS-T (1:250) and subsequently added to the prepared incubation rows in a 1:1 ratio; the protein concentration in each incubation well was halved in order to obtain a final serum dilution of 1:500. Reaction plates were incubated for 2 hours at 37 °C. Next, 60 µl of the TBS-T buffer were added to each well containing 40 µL of the serum solution. At the end of this step, each well contained 100 µL of serum that were diluted to 1:1250. Coated plates were washed three times with 300 µL/well of an ELISA washing solution. Plates were blocked and incubated at 37 °C for 2 hours. Blocked plates were washed 3 times with 300 µL/well of washing solution. Subsequently, 95 µL of prepared serum samples from incubation plates were transferred into the corresponding wells of the ELISA plate by means of a multichannel pipette. Experimental plates were covered and incubated at 37 °C for 1 hour. Next, the plates were washed as previously stated, and 100 µL/well of goat, anti-human IgG-Fc HRP-conjugated antibody (Bethyl Laboratories, Montgomery, TX, USA) was added. Plates were incubated at 37 °C for 1 hour. Following incubation, the plates were washed, and 100 µL/well of 3,3′,5,5′-tetramethylbenzidine (TMB) substrate (Bethyl Laboratories, Montgomery, TX, USA) was added and incubated in the dark at room temperature for 30 minutes. The reaction was stopped by adding 100 µL of an ELISA stop solution (Bethyl Laboratories, Montgomery, TX, USA), and then it was read within 20 minutes at 450 nm. Optical density (OD) values were used to draw a graph that confirmed the saturation of the samples with the protein (values reaching the lower plateau of the plot should have been seen in the samples that were treated with the highest concentration of the recombinant protein). Next, a blank OD was subtracted from all raw data results. The results from each serum sample in the three conditions (HA, head and no protein) were selected for stalk OD calculation. For these calculations, only 4 wells (at the lower plateau OD level) were used (e.g., the first four wells with the highest head (HA1) and HA protein concentrations). Results were calculated as follows:

$$\mathrm{OD}.\mathrm{HA}2=\bar{\mathrm{OD}}\mathrm{HA}1-\bar{\mathrm{OD}}\mathrm{HA}$$

$$\mathrm{OD}.\mathrm{HA}=\bar{\mathrm{OD}}\mathrm{NT}-\bar{\mathrm{OD}}\mathrm{HA}$$

$$\mathrm{OD}.\mathrm{HA}1=\left(\bar{\mathrm{OD}}\mathrm{NT}-\bar{\mathrm{OD}}\mathrm{HA}\right)-\left(\bar{\mathrm{OD}}\mathrm{HA}1-\bar{\mathrm{OD}}\mathrm{HA}\right)$$

Where $\bar{\mathrm{OD}}\mathrm{HA}1$ is the average OD of samples incubated with the head (HA1) protein, $\bar{\mathrm{OD}}\mathrm{HA}$ is the average OD of samples incubated with the HA protein, and $\bar{\mathrm{OD}}\mathrm{NT}$ is the average OD of samples incubated with the buffer (non-treated samples).

### 2.9. Statistical Analysis

Data were analyzed by GraphPad Prism. The ELLA, SRH and MN results were normalized by applying the Z-Score. Significant differences between pre- and post-vaccination OD signals (a value of 4 at the lower plateau level) for the head and stalk were evaluated with a paired T-test. The homogeneity of variances was previously verified through an F-test. A significance level of 5% was considered for all the statistical tests.

## 3. Results

### 3.1. Serum Samples Were Selected Based on HI Titers

**Figure 2:**
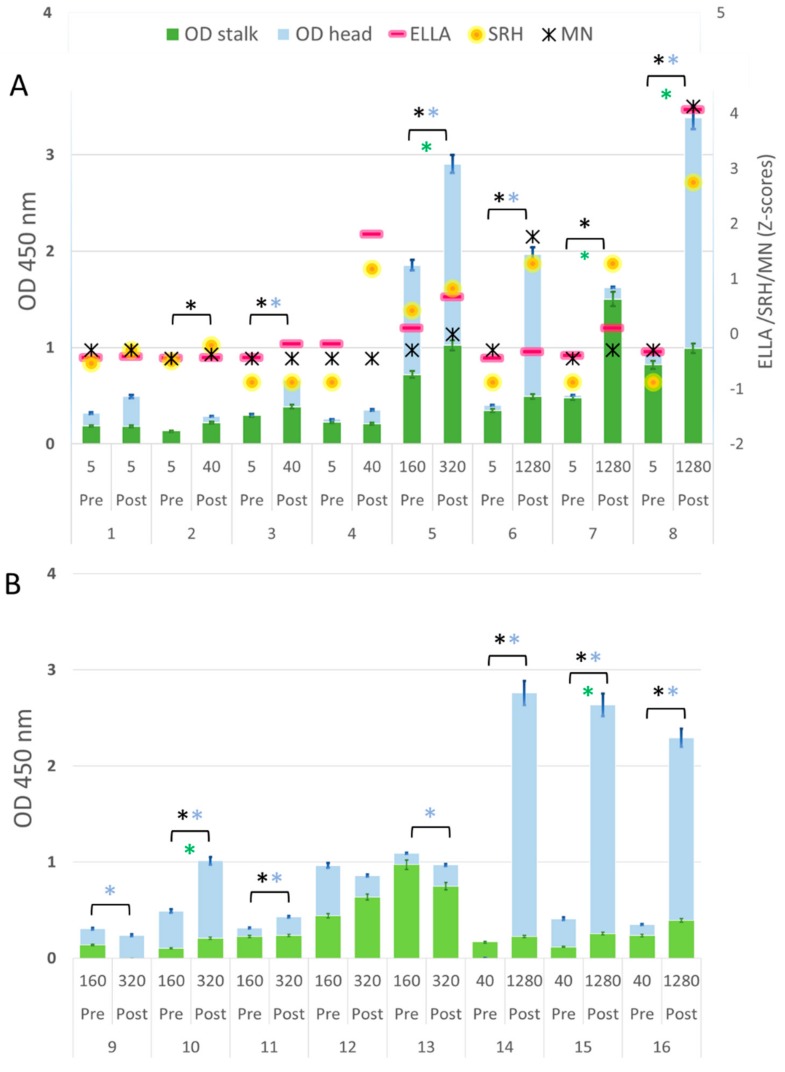
(**A**) Serum samples from adult subjects tested by ELISA, hemagglutination inhibition (HI), single radial hemolysis (SRH), micro-neutralization (MN) and enzyme-linked lectin (ELLA) assays; (**B**) Serum samples from adults tested by ELISA and HI assays. The HI titer of each sample is indicated below the x-axis. Blue bars = head signal, and green bars = stalk signal. Asterisks indicate statistical significance; a black asterisk indicates a significant increase in the HA signal; a blue asterisk indicates a significant increase in the head signal; a green asterisk indicates a significant increase in the stalk signal. Error standard bars are reported both for the head and stalk signals.

**Figure 3:**
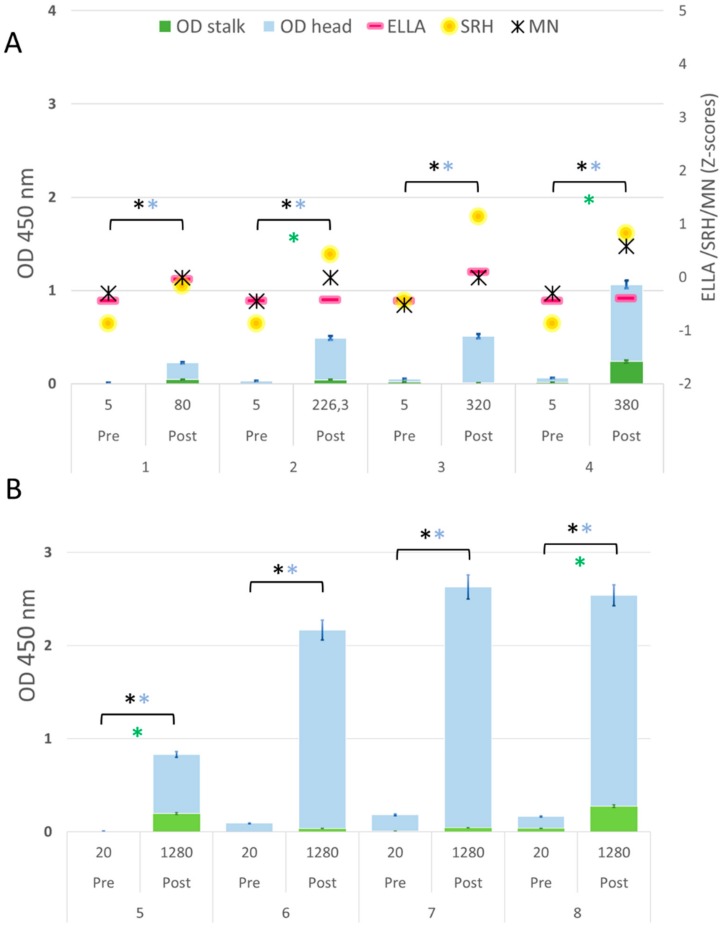
(**A**) Serum samples from children tested by ELISA, HI, SRH, MN and ELLA assays; (**B**) Serum samples from children tested by ELISA and HI. The HI titer of each sample is indicated below the x-axis. Blue bars = head signal, and green bars = stalk signal. Asterisks indicate statistical significance; a black asterisk indicates a significant increase in the HA signal; a blue asterisk indicates a significant increase in the head signal; a green asterisk indicates a significant increase in the stalk signal. Error standard bars are reported both for head and stalk signal.

Human serum samples that were obtained before and after vaccination were used in this study. As a proof-of-concept to evaluate the performance of ELISA in distinguishing between head- and stalk-specific differences, a total of 16 pairs of serum samples (pre-/post-vaccination) from adult subjects and eight pairs of serum samples (pre-/post-vaccination) from children were selected on the basis of their HI titers. In the first run of experiments, we selected eight adults and four children. Among the adults, we selected: one subject in whom the HI assay gave negative results both pre- and post-vaccination (5/5); three subjects with negative pre-vaccination HI titers and seroprotective post-vaccination HI titers (5/40); three subjects with very high boost (5/1280) of HI titers after vaccination; and one subject with a pre-existing HI titer of 160 which only marginally increased to 320 after vaccination. These selected samples were titrated by serological assays that are generally used in order to evaluate the immunogenicity of an influenza vaccine (MN, SRH and ELLA), along with the competitive head/stalk-specific ELISA described here (Table 1 and Figure 2A). The above-described serological analysis was repeated on the four samples from children, who had a pre-vaccination HI titer of 5 and post-vaccination HI titers of 80, 226.3, 320 and 380 (Table 2 and Figure 3A). We decided to investigate the immune response and the accuracy of the new ELISA method in a small number of children, too, because we expected to find significant differences in the stalk response between the two age-groups (adults and children) as a result of the previous exposure and/or vaccination of the adults. In the second run of experiments, to broaden our view of the variation in anti-head/stalk responses in individual subjects, we evaluated the performance of the head/stalk-specific ELISA on another eight pairs of samples from adults and four pairs of samples from children with different pre- and post-vaccination HI titers (Figure 2B and Figure 3B). 

### 3.2. Different Levels of Anti-HA2-specific Antibody Responses Were Found in Pre-Vaccination Samples from Adults, but Not in Children

We investigated the presence of anti-stalk antibody response in pre- and post-vaccination serum samples from adults and young children. All pre-vaccination samples from adults presented detectable stalk-specific antibodies (green bars in Figure 2A,B). The highest pre-vaccination levels assigned to the stalk antibodies were found in adult subjects 8 and 13, but they were completely independent from the measured HI titers of 5 and 160, respectively, which still correlated with the specific head response. In contrast to the results obtained in adults, no antibody responses against the stalk domain were detected in pre-vaccination pediatric serum samples when using the competitive ELISA, with the exception of subject 8 (Figure 3A,B); moreover, in both children and adults, the increased post-vaccination OD signal arose mainly from the head response (blue bars Figure 2 and Figure 3). Heterogeneous levels of anti-head antibody signals were detected in all samples; no head signals were found in samples 2 and 14 (adults) and sample 1 (children). The magnitude of the head response detected by ELISA after vaccination generally agreed with the increase that was registered by the HI assay, apart from three cases that were observed in adult subjects 1, 7 and 14. In subject 1, a post-vaccination increase in head antibodies was seen in the ELISA, but this was not seen in the HI assay, which remained negative after vaccination. Interestingly, we identified two adult subjects with positive HI titers of 1280 after vaccination (subject 7) or of 40 before vaccination (subject 14), though they had very low, or even undetectable head responses. These quite striking observations can most likely be attributed to the high level of antibodies against conserved epitopes, a response that may be able to result in an effective steric hindrance of hemagglutination activity.

### 3.3. Correlation between Anti-HA1 ELISA and SRH- and MN-Antibody Responses

Given that serological assays such as MN and SRH are not able to distinguish between antibodies against the head and stalk subunits of the HA, we compared the results yielded by the ELISA with those obtained with the aforementioned methods. The SRH data appeared to be more in line with the anti-head response detected by the ELISA and HI assay than with the titers measured by the MN assay. In all adults and children assessed by the SRH assay (yellow dot in Figure 2A and Figure 3A), we were able to detect a post-vaccination increase in the hemolysis area, apart from adult subject 3, who showed no increase in SRH but did show an HI seroconversion and an increase in the head response. By contrast, the MN assay seemed to be more specific than the SRH assay; it was possible to detect at least a two-fold increase in the neutralizing titer only in subjects that showed a greater increase in head response in the ELISA (adult subjects 5–8 and child subjects 1–4). For a more comprehensive overview on the immunological characterization of each subject, we also evaluated the anti-NA antibody response by using the ELLA test. We found that the NA response, as expected, generally did not correlate with the anti-head or the anti-stalk response. This was clearly seen in adult subjects 4 and 6. Subject 4 showed an eight-fold increase in ELLA but no increase in MN or stalk antibody responses and only quite low increases in HI and head responses. However, subject 6 did not show a post-vaccination increase in the NA antibody titer, despite high responses in the HI, MN and head ELISA tests. These results confirmed that the immunological responses against NA could not be related to or predicted by the HA responses.

## 4. Discussion

Along with vaccination coverage, which remains unsatisfactory [35], one of the main drawbacks of the current influenza vaccines is the need for an annual reformulation and consequent global re-administration, owing to the antigenic drift of the influenza virus. In the last two decades, growing interest in the possibility of developing a universal vaccine has given new impetus to influenza research. Several studies have focused on the extracellular domain of the M2 protein [36,37]. The ectodomain sequence has proven to be highly conserved among human and avian influenza viruses. However, antibodies that are elicited against this conserved portion are not neutralizing, but, due to the high expression of M2 on the surface of infected cells, they can promote protection through the effector function of their Fc region [38]. NA, the second most abundant glycoprotein that is present on the surface of the influenza virus, is another important target. Previous murine studies that were conducted with virus-like particles bearing the N1 antigen showed protection against lethal infection by homologous and heterologous strains [39]. Compared with the immunodominant globular head, the stalk domain is far less variable and is able to induce broadly neutralizing antibodies. The first description of a mouse monoclonal antibody that was specific for the stalk domain (C179) dates back to 1993 [40]. This antibody showed no HI activity; however, it was capable of neutralizing group 1 viruses (H1 and H2). In recent years, promising research has been carried out with a view to developing a stalk-based universal influenza vaccine; this research has mainly been based on a novel approach involving the construction of cHA molecules. Repeated vaccination with these constructs has been highly effective in boosting the antibody response against conserved regions of the stalk domain, resulting in high anti-stalk titers and a reduction of viral titers in lungs and nasal turbinates in mice and ferrets [16]. A universal influenza vaccine that is able to stimulate stalk-specific antibodies has the potential to avoid the need for the annual vaccine reformulation of the H1, H3 and B strains; moreover, it would confer greater protection against new emerging influenza viruses, particularly those that pose a pandemic threat [41]. In this paper, we present a possible approach that allows for head- and stalk-specific antibody responses to be clearly distinguished through the specific re-adaptation of a competitive ELISA. Unlike the HI, SRH, MN assays or the ELLA, which detect functional antibodies, this adapted ELISA only detects binding antibodies. Nevertheless, it can support studies of the immunogenicity of influenza vaccines by detecting and quantifying specific immune responses against mainly continuously changing epitopes in the head domain of the HA molecule (antigenic drift) and mainly conserved epitopes in the stalk region. This approach will be particularly helpful for the study of the immune responses that are induced by next-generation influenza vaccines, such as those based on conserved epitopes from the stalk domain of the HA protein [28,42,43].

The classical serological assays listed above are not able to detect and distinguish specific antibodies directed against the stalk region. Though, since February 2017, the new European Medicines Agency (EMA) guidelines have withdrawn the concept of the traditional correlates of protection for influenza, the HI titer is still considered the gold standard, and the correlates of protection based on this are still used in many countries, such as the U.S., Japan and Australia [44,45].

Here, we present the results obtained from a small number of samples selected on the basis of their HI titers. The ELISA IgG signal that was obtained against the HA protein agrees with those obtained with HI and SRH assays, for which we observed a better correlation (HI–SRH) (R^2^ = 0.70) to HI–MN and MN–SRH with R^2^ values of 0.55 and 0.3, respectively. Despite the low number of samples that were analyzed in the present work, these results seem to confirm previous studies that have supported strong agreement between the HI and SRH assays [46,47] in respect to the higher correlation found by Wang et al. [48] between the SRH and MN. The MN assay generally suffers from high interlaboratory variability due to the lack of common protocols (long vs short/CPE vs. ELISA-based) and discrepancies in endpoint determination. On the other hand, although ELISAs are not officially acknowledged by EMA and other regulatory authorities, they usually provide unbiased and precise results. The responses that were obtained in the two age groups support the statement that the described ELISA-based assay is able to distinguish between immunological responses against head and stalk epitopes in adults and children in a very selective manner. Moreover, inside each group (adults and children), the assay is able to reveal subtle differences in HA-specific responses. Upon comparing the results obtained in children and in adults, it appears that the immunological memory could play an important role in antibody responses after vaccination [49]. Indeed, in children, in whom we observed no or very low stalk signals, most of the response after vaccination was directed against the globular head domain. This particular ‘conserved’ trend that was observed in pediatric samples can be attributed to the low age of children (3–9 years) and the possibility for at least some of them of being completely naïve for A/H1N1/California/7/2009 influenza strain at the time of blood draw. The high anti-head antibody signal that was observed after vaccination in children, in contrast to the low anti-stalk signal, can be explained by different reasons: 1) the head domain of the HA is the most immunogenic part of the HA protein, in contrast to the stalk, which appears to be less immunogenic; 2) antibodies against the stalk domain are generally difficult to be elicited by classical inactivated split and subunit influenza vaccines [50]; and 3) the influenza specific B- (and T-) cells repertoires in young children contain a greater frequency of naïve cells. However, adults have pre-existing populations of influenza-specific memory cells that can target conserved epitopes [51]. This last point seems to validate our results from adult subjects, where we observed a boost in both head and stalk responses after vaccination and a more heterogeneous scenario in comparison to children. In one adult subject, we detected a particularly high anti-stalk response after vaccination and a very low anti-head response, despite a very high HI titer accompanied by increased SRH and NA responses. The unpredictability and complexity of immune responses against influenza vaccination are illustrated by the fact that this subject did not show an increase in the neutralizing antibody response. This peculiar observation can be explained by the interference of a large number of antibodies directed against conserved epitopes of the influenza virus, thus causing the steric hindrance of the hemagglutination activity. This was confirmed by the high SRH titer and the very low MN titer after vaccination, and it supports the superiority of the SRH assay over the HI and MN assays to detect a broader range of functional antibodies. This characteristic may not only reflect the specific nature of the SRH assay, which detects all antibodies directed against various epitopes of HA and NA, it may also reflect the fact that internal influenza virus proteins may be involved in the complement fixation reaction. The small increase in the MN titer, along with the low head response detected with the ELISA, seems to confirm this theory. This particular case supports the strategy that has been adopted in the last few years by some regulatory agencies, such as EMA [45,52], to take into account a combination of different immune responses that are measured by multiple assays for the evaluation of the effectiveness of influenza vaccines. In the present study, we also included the measurement of the NA antibodies in order to broaden the view of the antibody-mediated immune response in both age groups. However, it is important to point out that current licensed influenza vaccines are made with a well standardized amount of HA antigens but not of NA antigens. The understanding of the NA response could become extremely important for the study of the immune response after LAIV administration or natural infection.

## 5. Conclusions

In conclusion, the competitive ELISA described in here, when supported by parallel assays such as neutralization, SRH, NA inhibition and antibody-dependent cellular cytotoxicity reporter (ADCC), is able to accurately distinguish differences in individual immune responses, thereby allowing the mode of action of different (next-generation) influenza vaccine approaches to be interpreted. Specifically, as reported in several studies [26,53,54], the ADCC assay can reflect the functionality of the antibodies that are detected by ELISA. The results presented here confirm that the classical serological assays that are generally used to evaluate the immunogenicity of HA-based intramuscular/intradermal seasonal influenza vaccines are still valid. However, they could be insufficient in the evaluation of the immune response of next-generation influenza vaccines, especially if used alone.

This preliminary study presents some limitations, mainly based on the small number of samples that were analyzed and the use of HA and head subunits from a single influenza strain. Further studies will be done with the aim to qualify the assay, both by using a mixture of head and stalk reactive monoclonal antibodies as controls and by comparing the results obtained with other assays that are based on the use of chimeric HA proteins to directly detect stalk antibodies. Other parameters will address the influence of the protein concentration that is used during the treatment of samples and the inclusion of conformation-specific monoclonal antibodies to ensure that the head protein retains its native conformation after coating. In this first study, we used a quite high protein concentration in order to make sure that any serum antibodies were fully adsorbed or competed with soluble protein.

## Figures and Tables

**Table 1:** Samples from adult subjects tested by the HI, ELISA, ELLA, SRH and MN assays.

Subject	Dose	HI Titer	Competitive ELISA			ELLA Titer	SRH Area [mm2]	MN Titer
			OD Stalk (HA2)	OD Head (HA1)	OD HA			
1	Pre	5	0.187	0.133	0.320	10	10.2	40
	Post	5	0.182	0.295	0.477	15	17.3	40
2	Pre	5	0.133	−0.018	0.116	5	11.3	20
	Post	40	0.220	0.069	* 0.289	10	19.6	30
3	Pre	5	0.281	0.022	0.303	10	2.256	20
	Post	40	0.391	* 0.255	* 0.647	80	2.256	20
4	Pre	5	0.238	0.032	0.269	80	2.256	20
	Post	40	0.211	0.159	0.370	640	60.8	20
5	Pre	160	0.723	1.164	1.887	160	38.5	40
	Post	320	* 1.041	* 1.834	* 2.875	320	50.2	80
6	Pre	5	0.341	0.059	0.400	5	2.256	40
	Post	1280	0.485	* 1.464	* 1.949	40	63.6	320
7	Pre	5	0.452	0.039	0.491	20	2.256	20
	Post	1280	* 1.499	0.113	*1.612	160	63.6	40
8	Pre	5	0.816	0.131	0.947	40	2.256	40
	Post	1280	* 0.987	* 2.371	* 3.359	1280	107.5	640

In the ELLA, HI and MN assays, titers below 10 were assigned a value of 5 and considered negative. In an SRH assay, samples which did not show hemolysis were assigned an area value of 2.256 mm^2^. Statistically significant increases in OD stalk or OD head post-vaccination are marked with an asterisk.

**Table 2:** Samples from children tested by the HI, ELISA, ELLA, SRH and MN assays.

Subject	Dose	HI Titer	Competitive ELISA			ELLA Titer	SRH Area [mm2]	MN Titer
			OD Stalk (HA2)	OD Head (HA1)	OD HA			
1	Pre	5	0.006	0.007	0.014	5	2.256	40
	Post	80	0.039	* 0.179	* 0.219	120	21.2	80
2	Pre	5	−0.016	0.023	0.007	5	2.256	20
	Post	226.3	* 0.040	* 0.461	* 0.501	10	38.5	80
3	Pre	5	0.015	0.037	0.051	5	12.6	10
	Post	320	0.009	* 0.521	* 0.530	160	59.4	80
4	Pre	5	0.016	0.044	0.060	5	2.256	40
	Post	380	* 0.251	* 0.824	* 1.075	20	50.2	160

In the HI, ELLA and MN tests, titers below 10 were assigned a value of 5 and considered negative. In SRH, samples which did not show hemolysis were assigned an area value of 2.256 mm^2^. Statistically significant increases in OD stalk or OD head post-vaccination are marked with asterisk.
